# Inhibition of Aryl hydrocarbon receptor Interleukin-22 signaling and worsening of intestinal inflammation by *Sutterella* species

**DOI:** 10.1080/19490976.2026.2690688

**Published:** 2026-06-21

**Authors:** Louise Dupraz, Giovanna Orianne, Grégory Da Costa, Romain Gauthier, Amy Blondeau, Marie Boinet, Ambrîne Farfar, Laura Creusot, Aurélie Magniez, Léonard Dubois, Perle Guarino-Vignon, Loïc Chollet, Nathalie Rolhion, Camille Danne, Zdeněk Dvořák, Philippe Seksik, Olivier Berteau, Harry Sokol, Marie-Laure Michel

**Affiliations:** a Agro Paris Tech, Micalis Institute, Université Paris-Saclay, INRAE, Jouy-en-Josas, France; b Centre de Recherche Saint-Antoine, CRSA, AP-HP, Saint-Antoine Hospital, Gastroenterology Department, Sorbonne Université, INSERM, Paris, France; c Gut, Liver & Microbiome Research (GLIMMER), Fédération Hospitalo-Universitaire (FHU), Paris, France; d Department of Cell Biology and Genetics, Faculty of Science, Palacky University, Olomouc, Czech Republic

**Keywords:** IL-22, *Sutterella* sp., gut, AhR, IBD

## Abstract

The gut microbiota constitutes a complex ecosystem essential for host defense against infection and immune system maturation. Inflammatory bowel diseases (IBD), such as ulcerative colitis and Crohn's disease, are characterized by a severe inflammation of the intestine, arising from dysregulated control of host-microbiota crosstalk. However, neither the genetic bases of IBD nor the immune responses involved are fully understood. Pathobionts are currently under investigation for their active role in the development and severity of IBD. These bacteria are present in the gut microbiota of healthy individuals without causing disease, but have pathogenic potential when the intestinal environment is disturbed. Here, we highlight *Sutterella* sp. as a new commensal pathobiont for its capacity to modulate the host's immune functions. This anaerobic Gram-negative bacterium inhibits the production of IL-22 and IL-17 by lymphoid cells, *ex vivo* and *in vivo*. In the DSS-colitis model, *Sutterella sp.* can increase intestinal inflammation and inhibit IL-22 production. Moreover, the production of IL-22 and IL-17 by human lymphoid cells is also reduced by *Sutterella* sp. The bacterium acts directly on lymphoid cells through the secretion of protein-based compounds that antagonize AhR signaling. These data enhance our understanding of the mechanisms that regulate host immune functions and pave the way for new therapeutic strategies to control gut inflammation.

## Introduction

The gut microbiota forms a complex ecosystem that plays a crucial role in host defense against infections and immune system maturation.^1^ Inflammatory bowel diseases (IBD), such as ulcerative colitis (UC) and Crohn's disease (CD), are characterized by severe intestinal inflammation resulting from dysregulated interactions between the host and its microbiota.^1^ These pathologies require long-term treatments based on anti-inflammatory and immunosuppressive therapies, which are often associated with drug-related side effects. Moreover, some patients exhibit poor responses to existing therapies, highlighting the need for novel treatment approaches. Modulation of the gut microbiota is an actively studied strategy, notably through the use of probiotics, prebiotics, or fecal transplantation to enhance beneficial microbial species (such as *Faecalibacterium prausnitzii*) or microbiota-derived metabolites (such as short-chain fatty acids and aryl hydrocarbon receptor (AhR) ligands) that support gut health.^2^

The pathobionts are also currently under investigation for their active role in the development and severity of IBD.^3^ These bacteria are present in the microbiota of healthy individuals without causing disease, but can become pathogenic when the intestinal environment is disturbed. Research has primarily focused on adherent invasive *Escherichia coli* (AIEC), *Helicobacter hepaticus,* or *Ruminococcus gnavus.* ^3^ Identifying additional pathobionts and deciphering their mechanisms of action could lead to a better understanding and management of IBD pathophysiology and pave the way for a new generation of treatment.

Interleukin-22 (IL-22) is a member of IL-10 family, crucial to maintain epithelial barrier function, promote tissue repair, and protect against pathogens and pathobionts.^4^ ^,^ ^5^ This cytokine is produced by αβ CD4^+^ T helper 17 (Th17) cells, T helper 22 (Th22) cells, γδ T cells, and ROR-γt^+^ innate lymphoid cells (ILC3 CD4^+^ and CD4^neg^), in response to IL-23 and IL-1β produced by dendritic cells and macrophages. IL-22 targets mainly tissue epithelial cells that express IL-22R1, stimulates the production of protective mucus from goblet cells, and induces the production of antimicrobial peptides (RegIIIβ, RegIIIγ, and *β*-defensins).^6^ In mice, IL-22 protects the intestine against bacterial pathogens such as *Citrobacter rodentium* ^7^ and *Clostridioides difficile,* ^8^ but favors *Salmonella* *typhimurium* colonization^9^ and *Toxoplasma gondii*-induced immunopathology.^10^ During intestinal inflammation, IL-22 is present in high quantities in the blood of patients with Crohn's disease (CD).^11^ It protects the intestinal mucosa from inflammation *via* the maintenance of epithelial barrier integrity in CD.^12^ It also has a protective effect in the DSS (dextran sulfate sodium)-induced colitis mouse model.^13^ However, IL-22 also exacerbates inflammation in a chronic colitis model induced by the adoptive transfer of memory CD4^+^ T cells.^14^ IL-22 was thus shown to play beneficial or deleterious roles in different intestinal inflammation contexts, mainly depending on the cytokine environment. Considering its dual implication, understanding the interactions between the gut microbiota (especially the pathobionts) and IL-22-producing cells could identify promising targets for IBD therapy.

IL-22 expression in lymphoid cells is dependent on the transcription factor RORγt, but also and above all, on the aryl hydrocarbon receptor (AhR) and the transcription factor STAT3.^15-18^ Deficiency of AhR leads to decreased number of IL-22-producing CD3^neg^ and CD3^+^ cells in gut^19^ and AhR activation promotes IL-22 production by γδ T cells and ILC3.^17^ ^,^ ^20^ ^,^ ^21^ However, in the presence of IL-23, peripheral γδ T cells can still produce IL-22 in AhR KO mice.^22^ ^,^ ^23^ The activation and maintenance of IL-22-producing cells in the gastrointestinal tract depend on the gut microbiota since germ-free mice present an altered IL-22 compartment. While antibiotics reduce the production of this cytokine, colonization of antibiotic-treated mice with Clostridia upregulates IL-22 in colonic lamina propria.^24^ Several commensals and microbiota-derived metabolites have been shown to regulate IL-22-producing cells,^13^ ^,^ ^25-29^ including tryptophan metabolites,^13^ short-chain fatty acids^26^ ^,^ ^30^ and secondary bile acids.^31^ However, other mechanisms are at play.

Here, we identify *Sutterella* sp. as a new pathobiont belonging to the Pseudomonadota phylum, the Betaproteobacteria class, and the *Burkholderiales* order. This anaerobic Gram-negative bacterium directly inhibits IL-22 production by lymphoid cells *via* the secretion of a protein-based effector (>3 kDa) that antagonize aryl hydrocarbon receptor (AhR) signaling.

## Materials and methods

### Mice

C57Bl/6J mice (7–9-week-old females and males) were purchased from Janvier (France) and maintained under specific pathogen-free conditions. AhR KO on the C57BL/6JRj background and wild-type mice littermates were housed and bred at Saint Antoine Research Center. Animal experiments were performed according to the local ethical panel and the Ministère de l'Education Nationale, de l'Enseignement Supérieur et de la Recherche, France under agreement Apafis#20977-2019041215549049. All experiments were carried out using female mice, unless otherwise stated.

### Human samples

PBMCs were isolated from the peripheral blood of healthy donors obtained from Etablissement Français du Sang (EFS, convention n° 20/EFS/005).

### IBD cohort

The metagenomics analysis was performed on an internal cross-sectional cohort of French IBD patients (male = 346, female = 410) and healthy donors (male = 32, female = 62, NA = 4) ranging from 14 to 86 years old. Data regarding ancestry, race, and ethnicity were not recorded. Approval for human studies was obtained from the local ethics committee (Comité de Protection des Personnes Ile-de-France IV, IRB 00003835 Suivitheque study; registration number 2012/05NICB). Shotgun metagenomics data sequencing reads were filtered for low-quality (quality score Q < 20), fragmented reads (>75 bp), and sequencing adapters were removed using TrimGalore (version 0.6.10). Host reads were removed using a sensitive local alignment against the GRCh38 human genome with Bowtie 2. Taxonomic profiling was done using MetaPhlAn 4 with database version Jun23.^32^ Only samples with detectable *S. wadsworthensis* were included in the analysis. Analyzes were performed in R (v4.4.0), visualization was performed using ggplot2, ggpubr, and cowplot, with comparisons of relative abundance across clinical states assessed using first a Wilcoxon rank-sum test and adjusted for multiple comparisons using the Benjamini–Hochberg method, and last a linear model. The linear model used the following formula: log_abundance_SW ~ disease_state, history of surgery (ileocaecal resection, ileoanal anastasomosis), Antibiotics < 1 month, IBD duration, age, sex, Proton Pump Inhibitor intake + Biologics Treatment (for IBD) + Conventional immunosuppressant (for IBD), ileum involvement, number of reads. The model was significant (*p*-val = 0.003) with only three co-factors significantly contributing to the model: state—CD flare, number of reads, and IBD duration.

### Bacterial culture

*Escherichia coli* (K-12/MG1655), *Sutterella wadsworthensis* (DSM 14016), isolated from human abdominal fluid, and *Sutterella parvirubra* (DSM 19354), isolated from human feces, were grown at 37 °C in LYHBHI (BHI) medium [Brain–heart infusion medium supplemented with 0.5% (wt/vol) yeast extract (Difco, Le Pont De Claix, France) and 5 mg/L hemin] supplemented with Formate (1.8 mg/mL; Sigma-Aldrich St. Louis, MO, USA), Fumarate (1.8 mg/mL; Sigma-Aldrich), cysteine (0.5 mg/mL; Sigma-Aldrich), vitamin K1 (0.0001% (vol/vol), Sigma-Aldrich) and vitamin K3 (3.2 µg/mL, Sigma-Aldrich) in an anaerobic chamber (90% N_2_, 5% CO_2_, 5% H_2_). *Achromobacter denitrificans* (DSM 30026), isolated from soil, was grown at 37 °C in the same medium, with agitation in aerobic conditions.

Strains were cultured for 18 h, until the end of the exponential growth phase/beginning of the stationary growth phase. Cell concentration was measured with a flow cytometer (CytoFLEX, Beckman Coulter, Villepinte, France). After a centrifugation for 15 minutes at 4500 rpm, 4 °C, the supernatants are filtered through a 0.2 µm membrane and aliquoted for storage at −20 °C. Bacterial cell pellets were resuspended in PBS at a concentration of 1 × 10^9^ cells/mL, then heat-killed at 70 °C for 30 minutes. These suspensions were aliquoted for storage at −20 °C.

### Antibiotic and *Sutterella* treatments

In order to modulate the gut microbiota and create different scenarios, mice were treated with vancomycin (500 mg/L; Mylan, Canonsburg, PA, USA) and/or neomycin (1 mg/mL; Euromedex, Souffelweyersheim, France) in the drinking water and water solutions were prepared and changed every three days. Fluid intake was monitored and the antibiotic solution was changed every 3 d. In some experiments, mice were pre-treated 4 d with streptomycin sulfate salt (5 mg/mL, Sigma-Aldrich) in the drinking water in order to specifically deplete Proteobacteria from the gut microbiota and create a temporary ecological niche, before inoculating daily via intragastric gavage with a bacterial suspension of 10^9^ CFU/mL in 200 μL of PBS/glycerol or control medium (PBS/glycerol) for 3 weeks. In some other experiments, mice were inoculated daily via oral gavage with 200 μL of filtrated bacterial supernatant or control medium during 1 week.

### DSS treatment

To obtain IL-22-producing CD4^+^ αβ T cells from mesenteric lymph nodes, mice were administrated drinking water supplemented with 2% (wt/vol) DSS (MP Biomedicals, Solon, OH, USA) for 7 d, and then allowed to recover by drinking supplemented water for the next 2 d.

To study the impact of *Sutterella* on colitis, mice were inoculated daily via oral gavage with 200 μL of filtrated bacterial supernatant or control medium (BHI). One week after starting the gavages, mice were given 2% DSS dissolved in sterile drinking water *ad libitum* for 7 d, followed by a recovery period (water only) of 5 d. Animals were monitored daily for weight loss and disease activity index (including weight loss, stool consistency, and presence of blood in feces).

### Fractionation or treatment of *Sutterella* supernatants

Heat treatment at 90 °C for 35 minutes was performed. *S. wadsworthensis* culture supernatants and BHI medium were also treated with proteinase K (Qiagen, Hilden, Germany) overnight at 56 °C, and finally heated for 20 minutes at 96 °C to inactivate the enzyme. In some experiments *S. wadsworthensis* culture supernatants and BHI medium were fractionated by size using Amicon 3-kDa-molecular-weight-cutoff (MWCO) centrifugal filters. Filters were rinsed once with PBS 1% BSA (Sigma). Then, bacterial culture supernatants or BHI medium were added to the filter and centrifuged at 3000 g at 4 °C. The flow-throughs and the top fractions were collected.

### Fecal and ileal fluid DNA extraction

Fecal and ileal fluid DNA were extracted from the weighted samples as previously described.^13^

### Murine cell preparation

Murine cells were prepared as previously described.^30^ For isolation of mononuclear cells from mouse lamina propria, the SI between the stomach and cecum, large intestine between cecum and anal verge were cut out and open longitudinally. Tissues were washed with cold PBS1X to remove the intestinal content and cut cross-sectionally into 0.5–1 cm long pieces and then mixed with 5 mL pre-warmed buffer HBSS 1X (Gibco, Thermo Fisher Scientifics) with 5% (vol/vol) FCS (Eurobio Scientific, Les Ulis, France), 5 mM EDTA, 0.145  mg/mL dithiothreitol (DTT, Sigma-Aldrich), 10 mM HEPES (Gibco, Thermo Fisher Scientifics), 1% (vol/vol) penicillin‒streptomycin (P/S, Sigma-Aldrich) in the shaker at 37°C for 20 minutes. The supernatants were discarded and pellets were washed with cold PBS1X. The tissue pieces were then transferred to a new tube and digested with collagenase type IV (Gibco) or the mouse Lamina Propria Dissociation Kit (Miltenyi, Paris, France) and DNase (Roche, Bâle, Switzerland) for 30 minutes. All the contents were passed through a 100 μm cell strainer. LPLs were obtained using the 40/80 Percoll centrifugation (GE Healthcare, Chicago, IL, USA). For mouse lymph nodes preparation, peripheral lymph nodes (pLN, axillary, brachial, and inguinal) or mesenteric lymph nodes (mLN) were homogenized and washed in RPMI 1640 medium (Gibco) + 10% (vol/vol) FCS (Eurobio Scientific) + 1% (vol/vol) Hepes (Gibco) + 100 U/mL penicillin and 100 μg/mL streptomycin (Gibco).

### Human PBMCs isolation

As previously described,^30^ heparinized whole blood was diluted with PBS 1:1, layered over Histopaque-1077 (Sigma-Aldrich) and centrifuged at 400 g for 20 min without brake at room temperature. The peripheral blood mononuclear cell (PBMC) fraction was then washed in cold PBS and used for *in vitro* culture assays.

### *In vitro* culture assays and stimulation

Murine cells and human PBMCs were cultured in RPMI 1640 medium (Gibco) supplemented with 10% (vol/vol) FCS (Eurobio Scientific) + 1% (vol/vol) Hepes (Gibco) + 100 U/mL penicillin and 100 μg/ml streptomycin (Gibco) in the presence of BHI or *Sutterella* supernatant at 2, 5, or 10% or heat-killed *Sutterella* (MOI 10:1), during 4 or 24 hours. Murine cells were plated at 5 × 10^5^ cells (at 2.5 × 10^6^ cells/mL) per 96-well plate (CellStar) and stimulated for the 3 or 23 last hours in culture medium with 50 ng/mL phorbol 12-myristate 13-acetate (Sigma-Aldrich), 1 mg/mL ionomycin (Sigma-Aldrich), 20 ng/mL mouse IL-23 recombinant protein and 10 ng/mL mouse IL-1β recombinant protein (R&D Systems, Minneapolis, MN, USA). PBMCs were plated at 1 × 10^6^ cells per 24-well plate (CellStar) and stimulated 24 or 48 hours in culture medium with coated anti-human CD3 (2 μg/mL, Biolegend, San Diego, CA, USA) and soluble anti-human CD28 (4 μg/mL, Biolegend). The culture supernatants were frozen at −20 °C until processing. Cell viability was assessed with Zombie Aqua Fixable Viability Kit (BioLegend).

HT29 cell line (ATCC HTB-38) was cultured in Dulbecco's modified Eagle's medium (DMEM, Gibco) with glutamax supplemented with 10% (vol/vol) FCS (Sigma-Aldrich) + 100  U/mL penicillin and 100 μg/ml streptomycin (Gibco) at 37 °C in a 5% CO2 atmosphere. HT29 cells were plated (4 × 10^5^ cells per well), 72 hours before the experiments in a 24-well plate. For some experiments, cells were cultured with BHI or *Sutterella* supernatant and stimulated 24 hours with human recombinant TNF-*α* (5 ng/mL, Preprotech, Neuilly-sur-Seine, France). The culture supernatants were frozen at −20 °C until processing. For AhR activity analysis, cells were cultured 5 hours with BHI or *Sutterella* supernatant and stimulated for the 4 last hours with FICZ (6-Formylindolo[3,2-b]carbazole). Cell viability was confirmed using Invitrogen's Lactate Dehydrogenase (LDH) Colorimetric Activity Kit.

### Flow cytometry and cell sorting

Flow cytometry was carried out by using BD-LSR-Fortessa machine. αβ CD4^+^ T cells were sorted by using BD-Aria machine or mouse CD4^+^ T Cell Isolation Kit (Miltenyi).

Single-cell suspensions were prepared, as previously described,^16^ in FACS buffer (PBS + 2% (vol/vol) FCS + 0.01% (vol/vol) sodium azide; Sigma-Aldrich). Cells were stained on ice in PBS 1X (GIBCO) with Fixable Viability Dye eFluor 506 (eBioscience, San Diego, CA, USA) or Zombie Aqua Fixable Viability Kit (BioLegend). Cells were surface stained in FACS buffer with the following antibodies; from eBioscience: FITC–labeled anti-mouse CD3ε (145-2C11), PE-labeled anti-mouse CD4 (RM4-5), anti-CD16/32 (93), PECy7-labeled anti–mouse IFN-*γ* (XMG1.2), eF450-labeled anti-mouse IL-17 (17B7), PECy7-labeled anti-ROR-γT (B20), PE-labeled anti-human/mouse phospho-STAT3 (LUVNKLA); from BioLegend: APC-labeled anti-TCRγδ (GL3), PE-labeled anti-mouse IL-22 (Poly5164), BV785-labeled anti-mouse CD4 (RM4-5), BV605-labeled anti-mouse CD8α (53–6.7), BV711-labeled anti-human CD3 (UCHT1), AF700-labeled anti-human CD4 (RPA-T4), BV605-labeled anti-human CD8 (RPA-T8), Pacific Blue-labeled anti-human IL-17A (BL168), PECy7-labeled anti-human IL-22 (2G12A41). Cells were washed in FACS buffer before analysis. In mouse, γδ T cells are CD3^+^TCRγδ^+^ CD4^neg^ CD8^neg^, CD4^+^ T lymphocytes are CD3^+^TCRγδ^neg^ CD4^+^ CD8^neg^, CD4^+^ CD3^neg^ cells are γδ TCR^neg^ CD3^neg^ CD4^+^ CD8^neg^ and CD4^neg^ CD3^neg^ cells are γδ TCR^neg^ CD3^neg^ CD4^neg^ CD8^neg^. In human, CD4^+^ T lymphocytes are CD3^+^ CD4^+^ CD8^neg^.

For intracellular analysis of IL-17, IL-22, and IFN-*γ*, cells were first stimulated in the presence of 10 μg/mL Brefeldin-A (Sigma-Aldrich) for the 3 last hours (mouse) or 6 last hours (human). After surface staining, cells were fixed and permeabilized by using 4% (vol/vol) PFA in conjunction with 0.5% (wt/vol) saponin in PBS1X. We performed intracellular staining for ROR-γt according to the manufacturers' instructions (FOXP3/Transcription Factor Staining Buffer Set, Invitrogen, Carlsbad, CA, USA) and phospho-STAT3 according to the manufacturers' instructions (PermBuffer III, BD Bioscience, Franklin Lakes, NJ, USA). Data were analyzed by using FlowJo software (Tree Star Inc.), version 10.8.2.

### RNA expression analysis

As previously described,^30^ total RNA was isolated with RNeasy Micro Kit or Mini Kit (Qiagen) as per manufacturer's instructions. For real-time RT‒PCR analysis, RNA was reverse transcribed with LunaScript® RT SuperMix kit (New England Biolabs, NEB, Massachusetts, USA) by a mix of random hexamers and oligos-dT primers. Quantitative PCR was performed with Luna® Universal qPCR Master Mix (NEB) on StepOne equipment (Applied Biosystems, Foster City, CA, USA). Relative expression is displayed in arbitrary units normalized to GAPDH via the ΔΔCt method. Primer sequences are available upon request.

### Cytokine quantification

ELISAs were performed on the supernatants to quantify mouse IL-22 and IL-17A cytokines (Invitrogen), human IL-22, IL-17A, and IL-8 cytokines (R&D Systems), according to the manufacturer's instructions, with Tween 20 (Sigma-Aldrich) and H_2_SO_4_ (VWR Chemicals, Fontenay-sous-Bois, France). Legendplex (Mouse Th17 Panel, BioLegend) assay on cell supernatants was performed according to the manufacturer's recommendations. The results are normalized by cell viability measurement for each sample.

### Quantification of fecal lipocalin‑2 (LCN2) levels

Frozen fecal samples were weighed and suspended in cold PBS. Samples were then agitated on a Precellys (Bertin Corp., Montigny-le-Bretonneux, France) for 40 s at 6400 rpm using 4.5-mm glass beads to obtain a homogenous fecal suspension. Samples were then centrifuged for 5 minutes at 13,000 rpm (4 °C), and clear supernatants were collected and stored at −80 °C until analysis. LCN2 levels were estimated using a mouse Lipocalin-2/NGAL Duoset ELISA kit (R&D Systems) according to the manufacturer's instructions and expressed as pg/mg of stool.

### AhR activity measurement

The AhR activity was measured using HepG2-Lucia™ AhR reporter cells (InvivoGen, Toulouse, France) as described before.^13^ FICZ (6-Formylindolo[3,2-b]carbazole) was purchased from Enzo Life Sciences. Cell viability was confirmed using Lactate Dehydrogenase (LDH) Colorimetric Activity Kit (Promega, Madison, WI, USA).

## 16S sequencing

Bacterial DNA was extracted from the weighted stool samples or ileal fluids as previously described.^13^ More precisely, the feces samples were weighed and then resuspended in 4 M guanidine thiocyanate and *N*-lauroyl sarcosine (Sigma-Aldrich) and incubated at 70 °C for 1 hour. A mixture of 0.1- and 0.6-mm-diameter silica beads (VWR) was added, and the samples were shaken at 10,000 rpm three times for 20 s each in a Precellys (Bertin Technologies, Europe) apparatus. Polyvinylpolypyrrolidone was added to the tubes, which were then vortexed and centrifuged. After recovery of the supernatant, the pellets were washed with TENP (Tris-EDTA-NaCl, 1% polyvinylpolypyrrolidone) and centrifuged, and the new supernatant was added to the first supernatant. The washing step was repeated two times. The pooled supernatant was briefly centrifuged to remove particles and then split into two tubes. Nucleic acids were precipitated by the addition of 1 volume of isopropanol and centrifugation. Pellets were resuspended and pooled in phosphate buffer and potassium acetate. The tube was placed on ice overnight, and centrifuged. The supernatant was then transferred to a new tube containing RNase and incubated at 37 °C for 30 minutes. Nucleic acids were precipitated by the addition of sodium acetate and absolute ethanol. The tube was incubated at room temperature, and the nucleic acids were recovered by centrifugation. The DNA pellet was finally washed with 70% ethanol, dried, and resuspended in Tris–EDTA (TE) buffer. DNA suspensions were stored at −20 °C for real-time qPCR analysis of the 16S rDNA sequences.

16S rDNA gene sequencing was performed as previously described.^13^ Briefly, 16S rDNA gene PCR was performed using 5 ng genomic DNA according to the manufacturer's protocol (Metabiote) using 192 bar-coded primers (Metabiote MiSeq Primers). The PCR products were purified using an Agencourt AMPure XP-PCR Purification system (Beckman Coulter, Brea, CA, USA) and quantified according to the manufacturer's protocol. Sequencing was performed using a 300-bp paired-end sequencing protocol on an Illumina MiSeq platform (Illumina, San Diego, CA, USA) at GenoScreen, Lille, France.

### Scanning electron microscopy (SEM)

The SEM images were prepared as previously described.^33^ 50 µL of the bacterial culture were directly fixed in 2 mL volume of 2% glutaraldehyde buffered with sodium cacodylate 0.1 M, during 2 hours at room temperature, then overnight at 4 °C, in a 24-well plate (TPP 92024, Switzerland) that contains 1 cm × 1 cm glass slides at the bottom of the well. The glass slides were previously cleaned by 70% Ethanol, 10 minutes plasma cleaner, then cut with a diamond pen, sonicated 15 minutes in 100% Ethanol, then in MiliQ water, and coated with 0.01% Poly-L-lysin (Sigma Merck P4707-50 mL) during 5 minutes and finally rinsed with MiliQ water. Samples thus attached to the glass slides at the bottom of the wells were rinsed twice during 10 minutes in 0.2 M sodium cacodylate buffer, then in successive baths of ethanol (50, 70, 90, 100, and anhydrous 100%), and finally dried using a Leica EM300 critical point apparatus with slow 20 exchange cycles, and 2 minutes delay between steps. Samples were mounted on aluminum stubs with adhesive carbon (EMS, LFG France) and coated with 6 nm of Au/Pd using a Quorum SC7620, 50 Pa of Ar, 180 s of sputtering at 3.5 mA. Samples were observed using the SE detector of a FEG SEM Hitachi SU5000, 2 KeV, 30 spot size, 5 mm working distance. All the preparation steps were done using the MIMA2 core facility, INRAE, Jouy-en-Josas, France (https://doi.org/10.15454/1.5572348210007727E12).

### Radio-ligand binding assay

Ligand binding assay was performed using cytosolic protein extracts from murine Hepa1c1c7 cells, as previously described.^34^ Briefly, aliquots of protein (2 mg/mL) were incubated at the room temperature for 2 h with 2 nM [3H]-TCDD in the presence of DMSO (vehicle control), FICZ (positive control), 200 nM TCDF (non-specific binding), or increasing concentration of tested compounds. After the incubation, the hydroxyapatite slurry was added to the samples and the suspension was incubated on ice and washed three times with HEGT buffer. The hydroxyapatite pellet was re-suspended in scintillation cocktail, and radioactivity was determined in a liquid scintillation counter. The specific binding of [3H]-TCDD was determined by subtracting the radioactivity of nonspecific reaction (TCDF) from total radioactivity. The values of IC50 were calculated where appropriate.

### Quantification and statistical analysis

As previously described,^30^ results were expressed as the mean ± standard error of the mean (SEM). All statistical analyzes were performed using GraphPad Prism 10 software (San Diego, CA, USA), using a two-tailed Mann‒Whitney test or Wilcoxon test to compare two groups; and ordinary one-way ANOVA or a Friedman test to compare 3 groups. Correlation significance was determined using linear regression. *, *P* < 0.05; **, *P* < 0.01; ***, *P* < 0.001. *n* represents the number of mice or human donors per group. Statistical details of experiments and the exact value of *n* can be found in the figure legends.

## Results

### The abundance of *Sutterella* sp. bacteria correlates negatively with gut IL-22- and IL-17-producing cells

**Figure 1.**
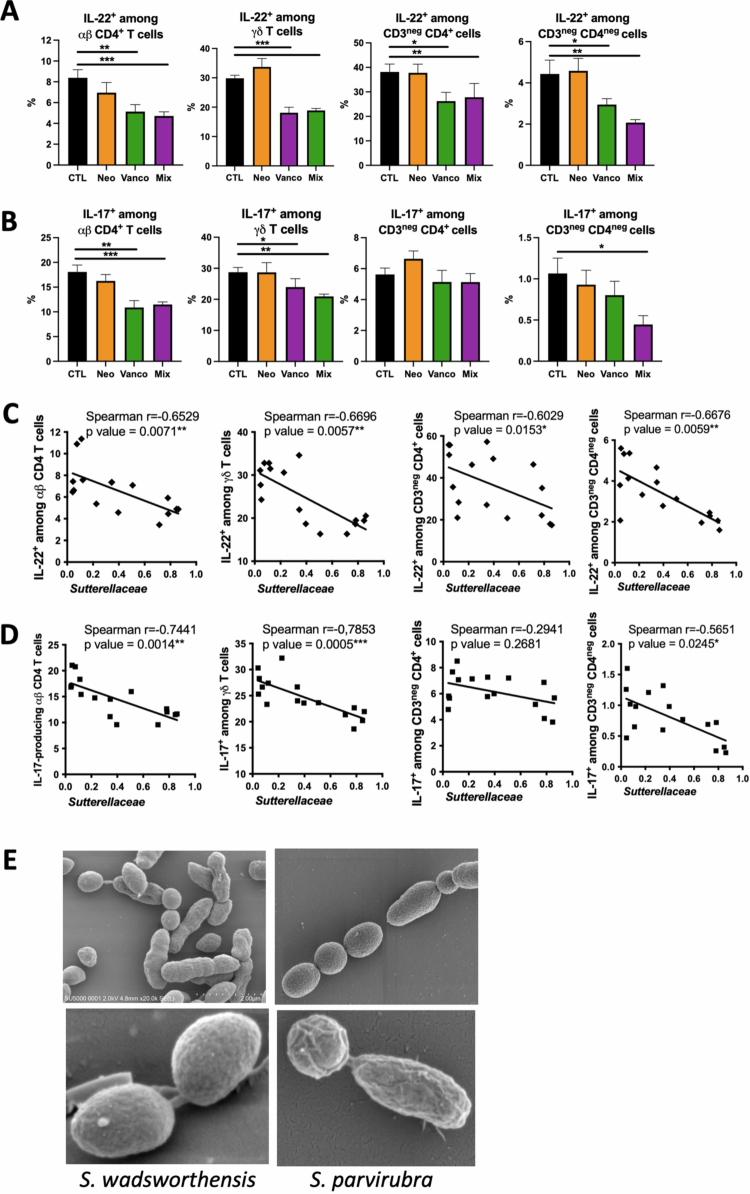
IL-22 and IL-17-producing cells reduce with antibiotics, while bacteria from *Sutterellaceae* family increase. (A, B) Percentage of IL-22 (A) and IL-17 (B) among gated CD4+ αβ T cells, γδ T cells, CD3^neg^ CD4^+^ and CD3^neg^ CD4^neg^ cells from small intestine obtained from untreated mice (control, CTL), neomycin (Neo)-, vancomycin (Vanco)– or neomycin + vancomycin (Mix)-treated mice. Cells were stimulated with PMA + ionomycin + IL-1β + IL-23 for 4 hours. (C, D) Correlation by Spearman r between percentage of IL-22 (C) and IL-17 (D) -producing cells gated as in A and *Sutterellacae* abondance in small intestine. (E) SEM images of *S. wadsworthensis* and *S. parvirubra*. Error bars are SEM from 5 to 10 mice per group (A, B) and from 16 mice (C, D). Significant differences were determined using Kruskal–Wallis test (A, B): *P < 0.05; **P < 0.01; ***P < 0.001. See also Supplementary Figure 1.

As previously reported,^30^ we found that vancomycin or a mix of antibiotics decreases the production of these two cytokines by αβ CD4^+^ T cells, γδ T cells, CD3^neg^ CD4^+^or CD3^neg^ CD4^neg^ cells (gated as described in Supplementary Figure 1A) in the murine small intestine (SI), and colon (Figure 1A and B; Supplementary Figure 1B and C). Only colonic γδ T cells are differentially regulated, as their production of IL-17 and IL-22 increased with antibiotic treatment (Supplementary Figure 1B and C), a result we previously observed.^30^

In order to generate different types of microbiome alterations, animals were treated with various antibiotics and microbiota composition were analyzed by 16S sequencing in the SI and colon of antibiotics-treated and untreated mice. This approach allowed us to create multiple microbiota configurations to investigate easier how variations in microbial composition influence IL-22 and/or IL-17 production. The proportion of IL-17^+^ and IL-22^+^ cells correlated positively with the abundance of segmented filamentous bacteria (SFB, *Candidatus arthromitus*) in the SI (Supplementary Figure 1D), which is in line with the well-described stimulatory effect of SFB on Th17 cells in the mouse SI,^25^ ^,^ ^29^ thus validating our approach. Interestingly, we also observed a negative correlation between IL-22 and IL-17 production and the relative abundance of bacteria from the *Sutterellaceae* family (Figure 1C and D; Supplementary Figure 1E and F), suggesting that members from this family could inhibit IL-22 and IL-17 production. Highly prevalent in the human intestinal mucosa, the genus *Sutterella* is known to be increased in the fecal microbiota of patients with UC,^35^ ^,^ ^36^ and is thus of interest in intestinal inflammation contexts. The two main species of *Sutterella* found in humans are *S. wadsworthensis* and *S. parvirubra*, with a mix of two different morphologies in pure culture: rod and oval shaped (Figure 1E).

### *Sutterella* sp. inhibits IL-22 and IL-17 production *in vitro*

**Figure 2.**
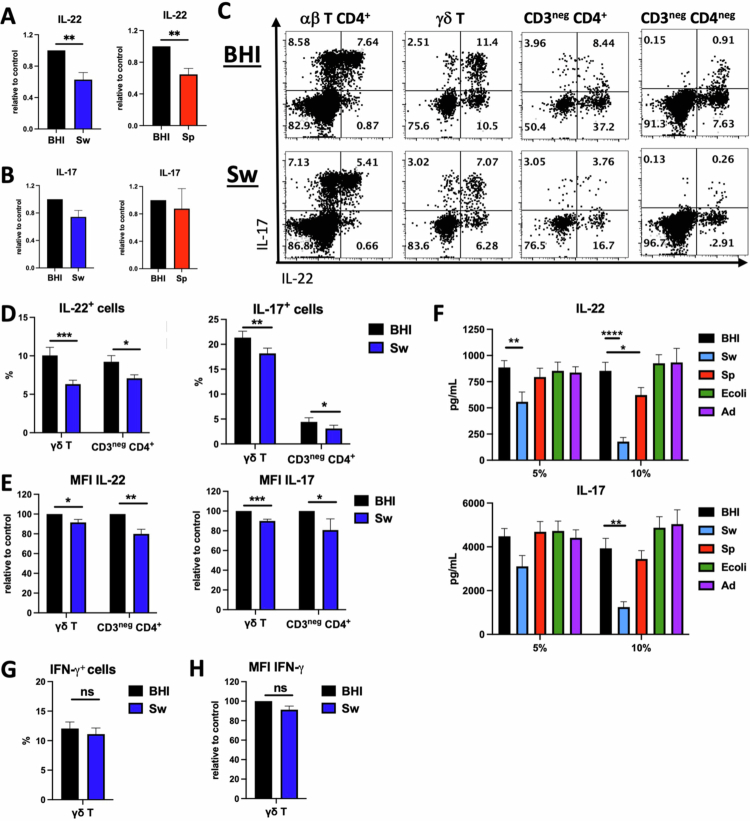
*Sutterella* regulates IL-17 and IL-22 productions in vitro. (A, B) Cells from LP of the small intestine were cultured for 24 h with BHI (black) or *S. wadsworthensis* (Sw, blue) or *S. parvirubra* (Sp, red) culture supernatant and stimulated with PMA + Ionomycin + IL-1β + IL-23. IL-22 (A) and IL-17 (B) productions were measured in the supernatants. (C) Intracellular analysis of IL-17 and IL-22 expression by gated αβ CD4^+^ T cells, γδ T cells, CD3^neg^ CD4^+^ cells, and CD3^neg^ CD4^neg^ cells from small intestine cultured with BHI (top), Sw (bottom) culture supernatant and stimulated for the 3 last hours with PMA + Ionomycin + IL-1β + IL-23. Data are representative of three independent experiments (D) Intracellular analysis of IL-22 (left) and IL-17 (right) expression by gated γδ T cells and CD3^neg^ CD4^+^ cells from pLN cultured with BHI (black), Sw (blue) culture supernatant and stimulated as in C. (E) Normalized geometric mean fluorescence intensity for IL-22 (left) and IL-17 (right) in gated γδ T cells and CD3^neg^ CD4^+^ cells obtained as described in D. (F) Cells from pLN were cultured for 24 h with BHI (black), Sw (blue), Sp (red), E. coli (Ecoli, green) or A. denitrificans (Ad, violet) culture supernatant and stimulated as in A. IL-22 (top) and IL-17 (bottom) productions were measured in the supernatants. (G) Intracellular analysis of IFN-*γ* expression by gated γδ T cells obtained as described in D. (H) Normalized geometric mean fluorescence intensity for IFN-*γ* in gated γδ T cells obtained as described in G. Error bars are SEM with 8–9 mice (A), 5 mice (B), 13 mice (D–F). ns, not significant, *P < 0.05, **P < 0.01, ***P < 0.001, ****P < 0.0001; by Wilcoxon test or Friedman test. See also Supplementary Figure 2.

To assess the effect of *Sutterella* sp. on IL-22 and IL-17 production, we used the culture supernatant of *S. wadsworthensis* and *S. parvirubra* to stimulate the immune cells isolated from the mouse SI lamina propria. *S. wadsworthensis* and *S. parvirubra* supernatants inhibited IL-22 production (Figure 2A) and, to a lesser extent, IL-17 production (Figure 2B) by murine immune cells *in vitro*. Flow cytometry analysis revealed that *S. wadsworthensis* inhibited IL-22 and IL-17 production by αβ CD4^+^ T cells, γδ T cells, CD3 ^neg^ CD4^+^or CD3 ^neg^ CD4^neg^ cells from SI, as early as 4 h (Figure 2C). To evaluate the generality of these observations, we analyzed cells from peripheral lymph nodes and found that the proportion and the quantity of IL-22 and IL-17 produced by peripheral γδ T cells and CD3^neg^ CD4^+^ cells, were also inhibited by *S. wadsworthensis* supernatants (Figure 2D and E), without inducing any toxicity (Supplementary Figure 2A). Within four hours of incubation, *S. parvirubra* only decreased the proportion of IL-22^+^ γδ T cells (Supplementary Figure 2B) without any toxic effects (Supplementary Figure 2C). Of note, culture supernatants of other members of the Pseudomonadota phylum (i.e. non-pathogenic *E. coli*), or Burkholderiales order (i.e. *Achromobacter denitrificans*), did not exhibit any inhibitory effect on IL-22 and IL-17 productions (Figure 2F). Moreover, there is no sex-based difference since IL-22 inhibition was observed in female (Figure 2D) and male mice (Supplementary Figure 2D).

Besides the inhibition of IL-17 and IL-22, *S. wadsworthensis* and *S. parvirubra* did not affect either the production of the pro-Th1 cytokine IFN-*γ* by peripheral γδ T (Figure 2G and H; Supplementary Figure 2E), or the release of IL-6, TNF-*α,* and IFN-*γ* by SI lamina propria cells (Supplementary Figure 2F). Finally, *Sutterella* did not alter IL-8 production by epithelial cell line, unstimulated or stimulated with TNF-*α*, in comparison to control (Supplementary Figure 2G).

### *Sutterella* sp. inhibits IL-22 production *in vivo*

In line with *in vitro* results, intragastric gavage of live *S. wadsworthensis* bacteria induced a significant reduction in the frequency of IL-22-producing CD3^neg^ CD4^+^cells in SI (Supplementary Figure 3A) and decreased production of IL-22 by all populations (αβ CD4^+^ T cells, γδ T cells, CD3^neg^ CD4^+^ and CD3^neg^ CD4^neg^) as demonstrated by geometric mean fluorescence intensity (MFI) analysis (Supplementary Figure 3B). Regarding IL-17, only its quantity produced by αβ CD4^+^ T cells was reduced in *S. wadsworthensis*-treated compared to control mice (Supplementary Figure 3C and D).

**Figure 3.**
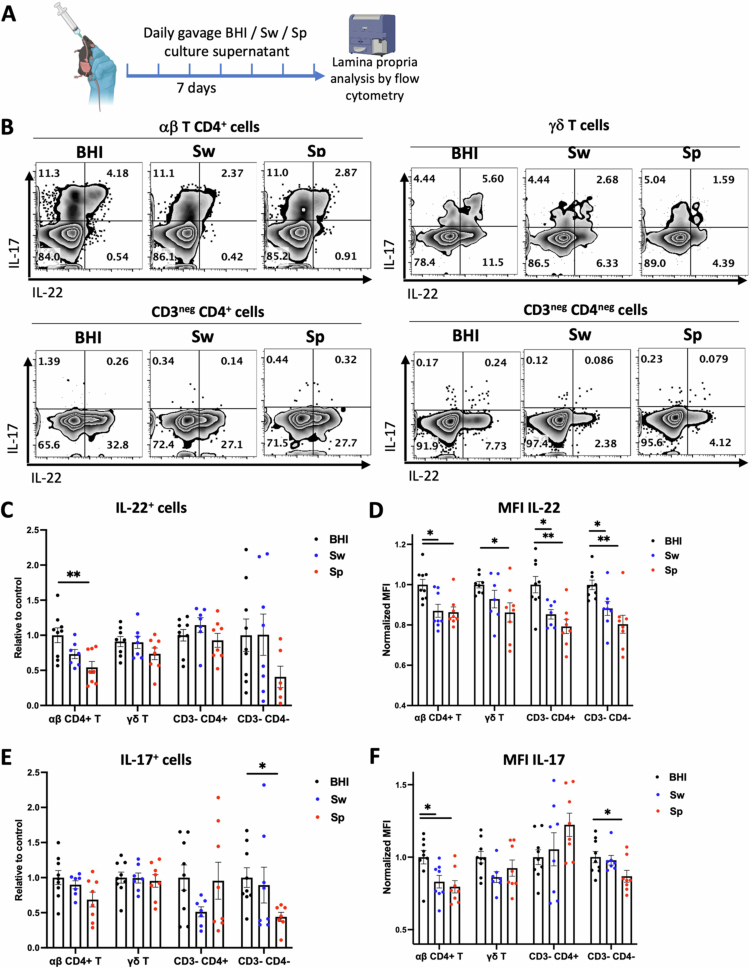
*Sutterella* regulates IL-17 and IL-22 productions *in vivo*. (A) Experimental design (B) Representative plots of gated αβ CD4^+^ T cells (top, left), γδ T cells (top, right), CD3^neg^ CD4^+^ (bottom left), CD3^neg^ CD4^neg^ (bottom right) cells from distal small intestine obtained from BHI (left), *S. wadsworthensis* (Sw, middle), or *S. parvirubra* (Sp, right) culture supernatant -treated mice. (C–F) Intracellular analysis of IL-22 (C) and IL-17 (E) expression and normalized geometric mean fluorescence intensity for IL-22 (D) and IL17 (F) by gated αβ CD4+ T cells, γδ T cells, CD3^neg^ CD4^+^, CD3^neg^ CD4^neg^ cells obtained as in B. In each case, cells were stimulated 3 hours with PMA + Ionomycin + IL-1β + IL-23. Error bars are SEM with 7–9 mice per group for each experiment. *P < 0.05, **P < 0.01, by Kruskal‒Wallis test. See also Supplementary Figure 3.

Because dead bacterial pellets have no effect *in vitro* on IL-17 and IL-22 production (Supplementary Figure 3E), we next analyzed the capacity of *Sutterella* culture supernatants from *S. wadsworthensis* and *S. parvirubra* to regulate these cytokines *in vivo* (Figure 3A). While the proportion of IL-22-producing αβ CD4^+^ T cells from distal SI and IL-22-producing CD3^neg^ CD4^+^ cells from proximal SI were reduced by intragastric gavage of culture supernatants (Figure 3B and C, Supplementary Figure 3F), the quantity of IL-22 produced was reduced for all populations (αβ CD4^+^ T cells, γδ T cells, CD3^neg^ CD4^+^ and CD3^neg^ CD4^neg^) from distal and proximal SI, in both strains-treated mice in comparison to control mice (Figure 3B and D; Supplementary Figure 3G). Thus, both species of *Sutterella* sp. are able to inhibit IL-22 production *in vitro* and *in vivo.* Concerning IL-17, as observed with live bacteria (Supplementary Figure 3C), the culture supernatants did not affect the proportion (Figure 3E, Supplementary Figure 3H) but reduced the quantity of cytokine produced by αβ CD4^+^ T cells from distal SI of *S. wadsworthensis* and *S. parvirubra*-treated mice (Figure 3F; Supplementary Figure 3I).

### *Sutterella* sp. increases intestinal inflammation and inhibits IL-22 production in the DSS-colitis model

**Figure 4.**
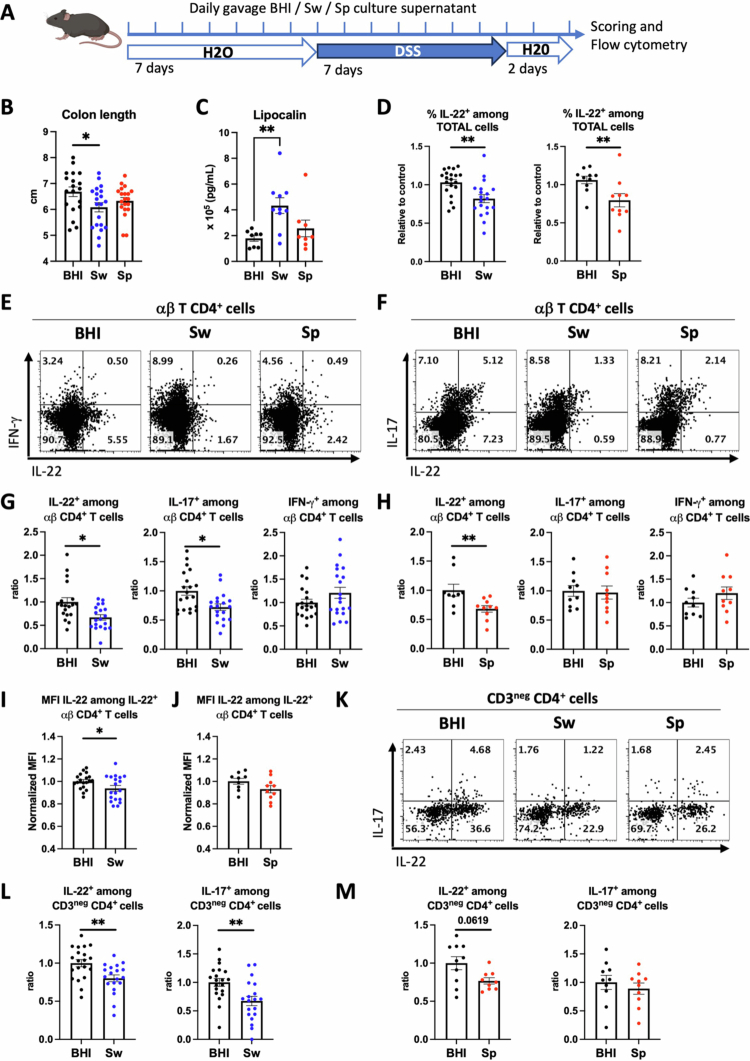
*Sutterella* culture supernatant increases intestinal inflammation and IL-17 and IL-22 productions in colitis. In all cases, mice were administrated with BHI, *S. wadsworthensis* (Sw) or *S. parvirubra* (Sp) culture supernatant and treated with DSS (A) Experimental design (B) Length of colon. (C) Lipocalin level in feces. Data represent a representative experiment from three independent experiments (D) Representative plots of total IL-22^+^ cells detected in colonic lamina propria stimulated for the 3 last hours with PMA + Ionomycin + IL-1β + IL-23. (E–J) Representative plots (E, F), intracellular analysis of IL-22, IFN-*γ,* and IL-17 expression (G, H) and normalized geometric mean fluorescence intensity for IL-22 and IL17 (I, J) produced by gated αβ CD4^+^ T cells, from colonic lamina propria stimulated for the 3 last hours with PMA + Ionomycin + IL-1β + IL-23. (K–M) Representative plots (K), intracellular analysis of IL-22 and IL-17 expression (L, M) by gated CD3^neg^ CD4^+^ cells obtained from colonic lamina propria stimulated for the 3 last hours with PMA + Ionomycin + IL-1β + IL-23. Error bars are SEM with 19–20 mice (B, G, I, and K), 8–9 mice (C) and 10 mice (D, H, J, and M) per group for each experiment. *P < 0.05, **P < 0.01, by Mann–Whitney test. See also Supplementary Figure 4.

To determine whether *in vivo* IL-22 repression by *Sutterella* sp. has an impact on mucosal injury, the bacterial supernatants were administered to mice submitted to DSS-induced colitis (Figure 4A). Our data revealed that culture supernatant of *Sutterella* sp. increased the severity of inflammation, as shown by a shorter colon (Figure 4B) and an increased fecal lipocalin level, a biomarker of intestinal inflammation (Figure 4C), without affecting the weight and disease activity index (DAI) curves (Supplementary Figure 4A). The total production of IL-22 by colonic lamina propria cells was also reduced in *S. wadsworthensis* and *S. parvirubra*-treated mice (Figure 4D). CD4^+^ αβ T cells and CD3^neg^ CD4^+^ cells, the main producers of IL-22 in the colon, were analyzed more specifically. The proportion of colonic IL-22-producing αβ CD4^+^ T cells was reduced by *S. wadsworthensis* and *S. parvirubra* culture supernatants (Figure 4E–H), while IFN-*γ*-producing αβ CD4^+^ T cells were increased (Figure 4E, G, and H). Only *S. wadsworthensis* culture supernatant reduced the proportion of IL-17-producing αβ CD4^+^ T cells (Figure 4F–H). The quantity of IL-22 produced by CD4^+^ αβ T cells was reduced in *S. wadsworthensis*-treated mice (Figure 4I and J), in contrast to IL-17 (Supplementary Figure 4B). Concerning CD3^neg^ CD4^+^ cells, the proportion of IL-22^+^ cells and the quantity of IL-22 were reduced in *S. wadsworthensis* and *S. parvirubra*-treated mice (Figure 4K–M, Supplementary Figure 4C and D). The percentage of IL-17^+^ cells among the CD3^neg^ CD4^+^ population was reduced by the administration of *S. wadsworthensis* only (Figure 4K–M, Supplementary Figure 4C and D). In summary, *Sutterella* sp. worsens mucosal injury and intestinal inflammation, and inhibits IL-22 production *in vivo* in a colitis context.

### *Sutterella* sp. inhibits IL-22 through AhR, independently of RORγt and STAT3

**Figure 5.**
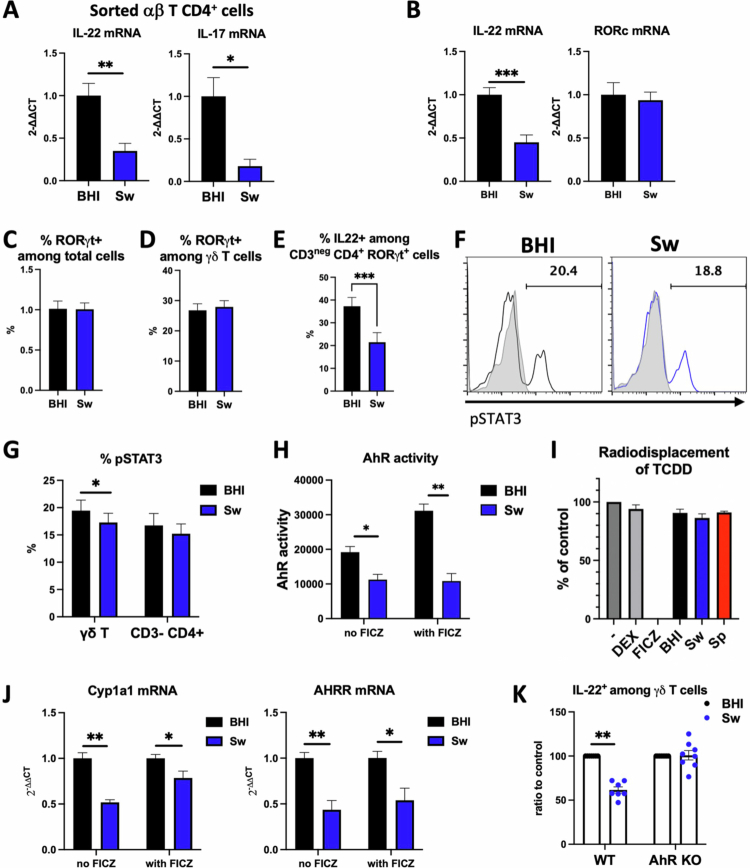
*Sutterella* regulates directly IL-22 producing cells by targeting AhR. (A) IL-22 mRNA quantification by qPCR in sorted αβ CD4^+^ T cells cultured 4 h with BHI (black) or *S. wadsworthensis* (Sw, blue) culture supernatant and stimulated with PMA + Ionomycin + IL-1β + IL-23 for the 3 last hours. Mice were treated with DSS and αβ CD4^+^ T cells were sorted from mLN. (B) Cells from pLN were cultured and stimulated as in a. IL-22 (left) and RORc (right) mRNA were quantified by qPCR. (C, D) Proportion of ROR-γt^+^ cells among total cells (c) or γδ T cells (d) from LN cells cultured and stimulated as in A. (E) Proportion of IL-22^+^ cells among ROR-γt^+^ CD3^neg^ CD4^+^ cells from LN cells cultured and stimulated as in A. (F) Flow cytometric detection of intracellular pSTAT3 in gated γδ T cells from pLN, cultured 2 h with BHI or Sw (blue) culture supernatant and stimulated with IL-23 for the last hour. Open and shaded areas indicate IL-23 treatment and controls, respectively. (G) Proportions of pSTAT3^+^ cells among γδ T cells and CD3^neg^ CD4^+^ cells from pLN, cultured as in F. (H) AhR reporter cell line activation without or with FICZ, in presence of BHI (black) or Sw (blue) culture supernatant. Data represent a representative experiment from 3 independent experiments. (I) Dexamethasone (negative control), FICZ (positive control), BHI (black) Sw (blue) or Sp (red) culture supernatant were tested for their ability to displace [^3^H] TCDD. (J) Adherent HT29 cells were cultured for 5 hours with BHI (black) or Sw (blue) culture supernatant and stimulated with FICZ for the 4 last hours. Cyp1a1 (left) and AHRR (right) mRNA were quantified by qPCR. (K) Intracellular analysis of IL-22 expression by gated γδ T cells from pLN of WT and AhR KO mice, cultured and stimulated as in A. The Sw-treated cells from WT and AhR KO mice are normalized against their own control BHI). Error bars are SEM with 7–11 mice (A–E), 7–8 mice (G), 5 biological replicates (H, I), 6 biological replicates (J) and 5–6 mice (K). *P < 0.05, **P < 0.01, ***P < 0.001; by Wilcoxon test. See also Supplementary Figure 5.

To assess the direct effect of *Sutterella* supernatants, sorted αβ CD4^+^ T cells were examined. Upon stimulation, the induced production of IL-22 and IL-17 was inhibited by *S. wadsworthensis* and *S. parvirubra* culture supernatants (Figure 5A and Supplementary Figure 5A). Thus, *Sutterella* effectively inhibits αβ CD4^+^ T cells functions through direct effects.

The expression of the transcription factor ROR-γt was assessed after stimulation of pLN cells with *S. wadsworthensis* and *S. parvirubra* culture supernatants. Although we observed an inhibition of IL-22 mRNA expression following incubation with *Sutterella* supernatants, the expression of RORc gene was maintained (Figure 5B, Supplementary Figure 5B). The proportion of total ROR-γt^+^ cells was not altered by *S. wadsworthensis* and *S. parvirubra* culture supernatants (Figure 5C, Supplementary Figure 5C), as also observed at the γδ T lymphocyte level (Figure 5D, Supplementary Figure 5D). Moreover, among RORγt^+^ ILC3 (CD3^neg^ CD4^+^ RORγt^+^ cells), *S. wadsworthensis* and *S. parvirubra* culture supernatants reduced the proportion of IL-22-producing cells (Figure 5E, Supplementary Figure 5E). Therefore, *Sutterella* sp. acts on IL-22 and IL-17 production independently of RORγt inhibition.

Concerning the factor of transcription STAT3, its IL-23-dependent phosphorylation was comparable among cells incubated with the control medium or the supernatants of *S. wadsworthensis* and *S. parvirubra* (Figure 5F and G; and Supplementary Figure 5F and G), excluding the involvement of STAT3 in the effect of *Sutterella* sp.

Using an AhR reporter system, we found that *S. wadsworthensis* culture supernatants reduced AhR activation (Figure 5H). We next performed a radioligand displacement assay to investigate the capacity of *Sutterella* to directly block AhR binding. Our data showed that *Sutterella* sp. effectors modulate the AhR signaling pathway, acting either upstream or downstream of AhR since *S. wadsworthensis* supernatants did not inhibit the binding of ^3^H-TCDD to AhR (Figure 5I). *S. wadsworthensis* culture supernatant hinders the cellular functions of AhR by downregulating AhR ligand-inducible genes such as Cyp1a1 and AhRR (AhR repressor) (Figure 5J). Despite the drastic decrease of total γδ T cells and IL-22-produing γδ T cells in small intestine of AhR KO in comparison to littermates (Supplementary Figure 5H-I), γδ T cells are maintained in pLN of AhR KO (Supplementary Figure 5J) and can produce IL-22 upon IL-23 and IL-1β stimulation (Figure 5K, Supplementary Figure 5K). Therefore, using this organ, we could study the impact of *Sutterella* on γδ T cells via AhR pathway. Among this population, we found that *S. wadsworthensis* also inhibits IL-22 production through AhR since its culture supernatant failed to reduce *ex vivo* IL-22 produced by cells from AhR KO mice (Figure 5K).

Concerning *S. parvirubra*, in parallel to its more moderate effect on IL-22 *in vitro* and *in vivo*, its dependence on AhR is weaker (Supplementary Figure 5L and M).

### *Sutterella* sp. inhibits human IL-22 and IL-17

**Figure 6.**
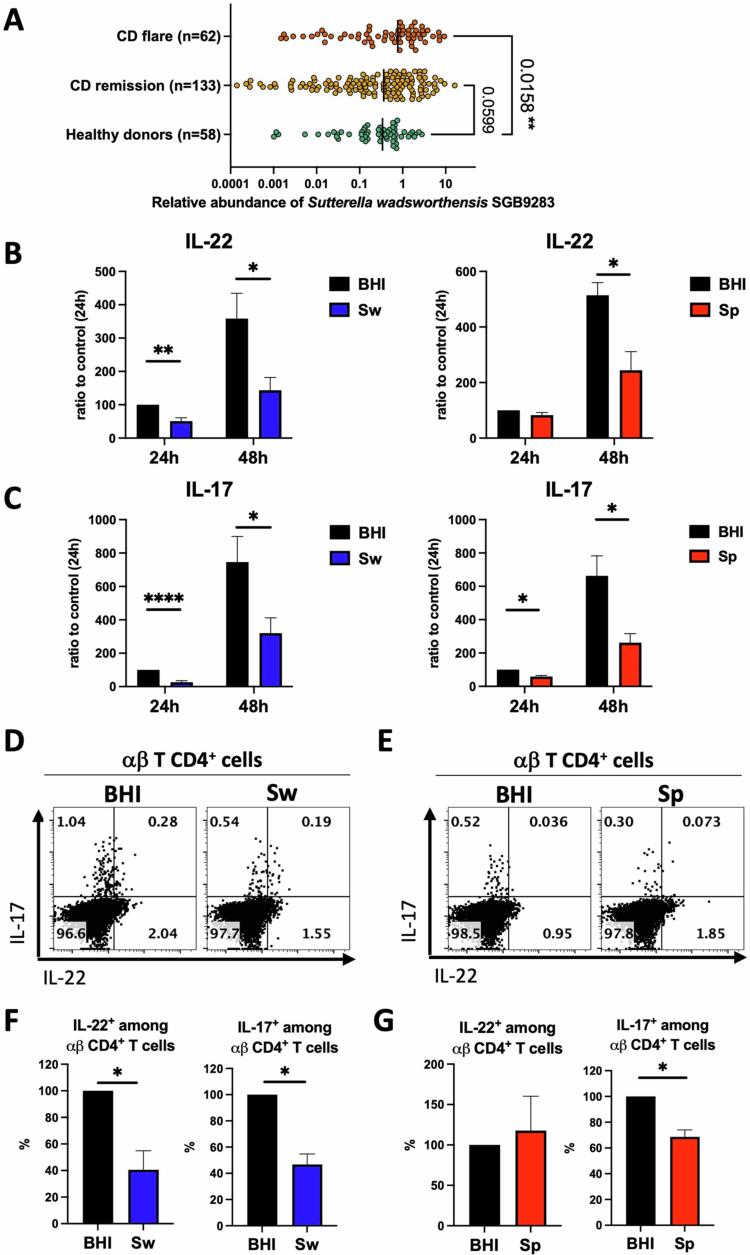
*Sutterella* regulates IL-22 and IL-17 productions in human. (A) Comparison of the relative abundance of *S. wadsworthensis* SGB9283 using MetaPhlAn 4 taxonomic profiling in an internal cross-sectional Crohn disease cohort. (Wilcoxon rank-sums tests Padj < 0.05, see Methods). (B, C) Cells from peripheral blood mononuclear cells (PBMCs) were cultured for 24 or 48 hours with BHI (black), *S. wadsworthensis* (Sw, blue), or *S. parvirubra* (Sp, red) culture supernatant and stimulated with anti-CD3 and anti-CD28. IL-22 (B) and IL-17 (C) productions were measured in the supernatants. (D–G) Intracellular analysis of IL-17 and IL-22 expression by gated αβ CD4^+^ T cells from PBMC cultured 24 hours with BHI, Sw (D, F) or Sp (E, G) culture supernatant and stimulated with anti-CD3 and anti-CD28. Error bars are SEM from *n* = 7–9 donors (B, C) and 5–6 donors (D–G). Significant differences were determined using the Wilcoxon test, *P < 0.05, **P < 0.01, ****P < 0.0001 See also Supplementary Figure 6.

We observed in a cohort of patients with Crohn's disease that patients in flare exhibit higher relative abundance of *S. wadsworthensis* compared to healthy donors (Figure 6A) and this even while taking into account the clinical background of patients (linear model with all co-variates: *p*-val = 0.003, CD flare against healthy covariate: estimate = 0.38 et *P*-val = 0.03). These findings highlight the significant relevance of our data in human contexts, especially since a reduced number of IL-22-producing cells in actively inflamed tissues has been shown, suggesting a link between *Sutterella*, IL-22, and IBD in patients.

We next treated human peripheral blood mononuclear cells (PBMCs) with *S. wadsworthensis* and *S. parvirubra* supernatants for 24 and 48 hours and measured the production of IL-22 and IL-17. As in mice, *Sutterella* inhibits the production of human IL-22 and IL-17 (Figure 6B and C). Of note, the inhibition of IL-22 by *S. parvirubra* culture supernatant was observed at 48 hours and not at 24 hours, suggesting a more moderate and/or slower effect of this strain in comparison to *S. wadsworthensis.* These results were confirmed by flow cytometry since the proportion of IL-22 and IL-17-producing αβ CD4^+^ T cells was reduced after 24 hours of incubation with *S. wadsworthensis* culture supernatant (Figure 6D–G).

### A proteinaceous effector produced by *S. wadsworthensis* inhibits mouse and human IL-22

**Figure 7.**
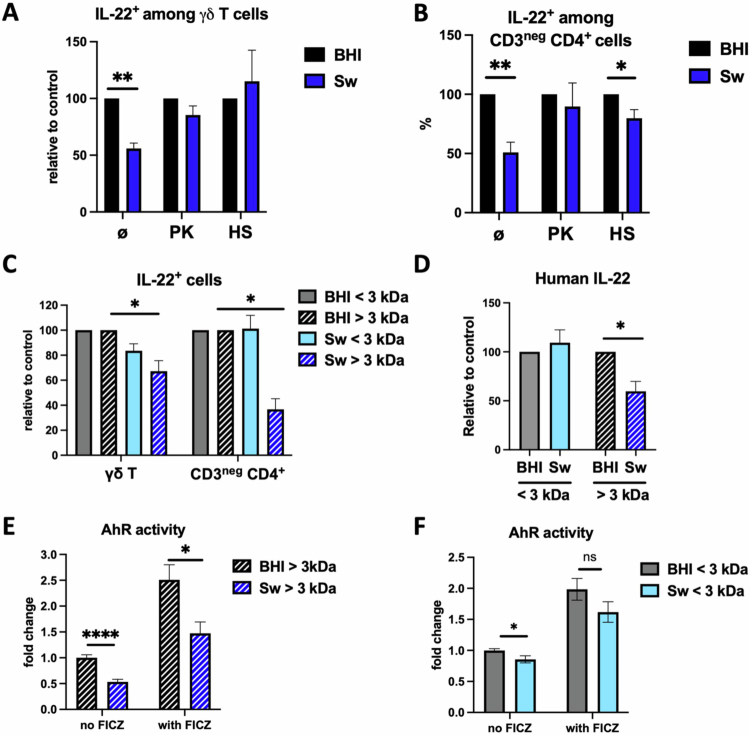
A >3 kDa protein from Sutterella regulates IL-22 production. (A, B) Intracellular analysis of IL-22 expression by gated γδ T cells (A) and CD3neg CD4+ cells (B) from pLN cultured 4 hours with BHI or Sw culture supernatant (pretreated with proteinase K (PK) and/or pre-heated (Heat shock, HS) and stimulated with PMA + Ionomycin + IL-1β + IL-23 for the 3 last hours. (C) Intracellular analysis of IL-22 expression by gated γδ T cells and CD3^neg^ CD4^+^ cells from pLN cultured 4 hours with <3 kDa and >3 kDa fractions of BHI or S. wadsworthensis culture supernatant and stimulated as in A. (D) Cells from peripheral blood mononuclear cells (PBMCs) were cultured for 24 hours with BHI (black), *S. wadsworthensis* (Sw, blue) culture supernatant and stimulated with anti-CD3 and anti-CD28. IL-22 production was measured in the supernatants. (E, F) AhR reporter cell line activation without or with FICZ, in presence of >3 kDa (E) and <3 kDa (F) fractions of BHI or *S. wadsworthensis* culture supernatant. Error bars are SEM from 8 mice (A, B), 8–13 mice (C), 6 donors (D) 8–11 biological replicates (E, F). Significant differences were determined using the Wilcoxon test (A-C), paired t test (C) and Mann–Whitney test (E-F). *P < 0.05, **P < 0.005. See also Supplementary Figure 7.

The *Sutterella* effectors responsible for inhibiting IL-22 and IL-17 are secreted molecules, as evidenced by the repressor effect of the culture supernatant (Figure 2), unlike bacterial cell wall components (Supplementary Figure 3E). To assess the nature of this/these effectors, the supernatants were heat-treated, proteolyzed by proteinase K, and/or filtered using different molecular weight cutoff membranes. These various treated supernatants did not affect murine and human cell viability (Supplementary Figure 7). *S. wadsworthensis* culture supernatants heated and treated with proteinase K lost their inhibitory effect on IL-22 production (Figure 7A and B). Moreover, the <3 kDa fraction had no effect on IL-22 production by γδ T cells, CD3^neg^ CD4^+^ cells, while the >3 kDa fraction inhibited this cytokine (Figure 7C). The inhibition of human IL-22 produced by PBMC was also observed only with the >3 kDa fraction of *S. wadsworthensis* culture supernatant (Figure 7D). Of note, this fraction could significantly reduce the activation of the AhR pathway in the absence and presence of FICZ (Figure 7E), while the <3 kDa molecules from *S. wadsworthensis* culture supernatant have only a very minor effect on AhR in the absence of FICZ (Figure 7F). Taken together, these results support that the effectors of *S. wadsworthensis* activity are not metabolites (as shown for other bacterial commensals) but >3 kDa proteinaceous compounds, likely surface proteins.

## Discussion

In this study, we observed that antibiotic treatment increases the proportion of betaproteobacteria from the *Sutterellaceae* family, which correlates negatively with the proportion of IL-22 and IL-17-producing cells in the murine SI and colon. *In vitro* and *in vivo* analyzes then showed that two species from this bacterial family, *S. wadsworthensis* and *S. parvirubra*, are capable of inhibiting these two cytokines produced by αβ CD4^+^ T cells, γδ T cells, CD4^+^ CD3 ^neg^ or CD4^neg^ CD3 ^neg^ cells. These findings are relevant in humans since *Sutterella* sp. also reduces the level of IL-22 and IL-17 secreted by human PBMCs.

The regulation of IL-22 production is primarily associated with gut microbiota-derived metabolites, such as tryptophan metabolites,^13^ short-chain fatty acids,^26^ ^,^ ^30^ and secondary bile acids.^31^ ^,^ ^37^ Here, we demonstrated that the bio-active effectors of *Sutterella* sp. are >3 kDa proteinaceous molecules that act directly on lymphoid cells. On the host side, the production of IL-22 can be regulated through several signaling pathways. IL-23 signal is primarily transduced by STAT3.^16^ However, IL-23-dependent STAT3 phosphorylation was comparable between cells incubated with *Sutterella* culture supernatant and culture medium, suggesting that the bio-active effectors of *Sutterella* sp. do not prevent IL-23 from binding to its receptor and may not directly inhibit the JAK/STAT3 cascade, the canonical IL-23 signaling pathway.

AhR is another major transcription factor, essential for IL-22 production by ILC3,^15^ Th17 cells^16^ and γδ T cells.^17^ It can modulate Th17 development independently of ROR-γt expression and STAT3 phosphorylation.^38^ However, AhR does not act alone but cooperates with other transcription factors to induce the production of IL-17 or IL-22. AhR facilitates notably ROR-γt recruitment at the IL-22 locus to induce its expression.^39^ In line with this, our data suggest that *Sutterella* sp. inhibits IL-22 and IL-17 production by reducing AhR activation without affecting ROR-γt expression. While low-affinity microbially produced indoles can compete with the high-potency agonist TCDD,^40^ ^,^ ^41^ the *Sutterella* culture supernatant did not displace 3H-TCDD from the AhR. Rather, we can speculate that the bio-active effectors of *Sutterella sp.* inactivate or degrade AhR agonists present in BHI medium or interfere directly with AhR signaling or indirectly with factors affecting AhR, such as ARNT, hsp90, and PKC. Therefore, future analysis will be necessary to understand how they control AhR activation, with a special focus on nucleus translocation and heterodimerization, post-transcriptional regulation of AhR targets and crosstalk of AhR with key signaling pathways. Additional pathways could also be investigated such as MAPK/ERK or PI3K, that are known to contribute to IL-23-mediated IL-22 production.^42^ *Bacillus anthracis* is a bacterium capable of suppressing IL-22 production in ILC3 which disrupts the MAPK pathway—specifically the MEK/ERK and MKK3/6-p38 cascades while leaving the JAK-STAT pathway largely intact.^43^ Therefore, MAPK signaling is a potential pathway by which *Sutterella sp*. could regulate IL-23-driven IL-22 expression.

*Sutterella sp.* is an anaerobic and microaerophilic Gram-negative bacterium, highly prevalent in the human intestinal mucosa, and more abundant in patients with IBD,^44^ autism^45^ or metabolic syndrome.^46^ Children with autism who have gastrointestinal dysfunction are more frequently *Sutterella*-positive.^45^ Despite their abundance in the human intestinal mucosa,^36^ the interactions between *Sutterella sp* and the host have been poorly characterized. Previous studies showed positive and negative correlations of *Sutterella sp* with pro-inflammatory cytokines,^47^ ^,^ ^48^ bringing a contentious role of this bacterium in immune regulation. *Sutterella* sp. can adhere to intestinal epithelial cells,^36^ but the current study is the first evidence of the direct effect of *Sutterella* sp. on immune cells. Although it has been shown that *S. wadsworthensis* has a mild pro-inflammatory activity on epithelial cells,^36^ we did not observe the induction of IL-8 by epithelial cells or a pro-Th1 cytokine profile in immune cells stimulated with *Sutterella* sp, but instead a decrease of IL-22 and IL-17 production. These two cytokines have a dual role in gut inflammation, since they were shown to play beneficial^12^ ^,^ ^13^ or deleterious^14^ roles in gut inflammation contexts, mainly depending on the cytokine environment. IL-22 shows a protective role in the mouse model of colitis induced by DSS^13^ but contributes to increased inflammation in the adoptive transfer of memory CD4⁺ T cells.^14^ Concerning IL-17, it can contribute to inflammation but also plays an important role in maintaining intestinal epithelial homeostasis and mucosal barrier integrity. Experimental and clinical studies show that blocking IL-17 can worsen DSS-induced colitis^49^ and increase activity in Crohn's disease,^50^ suggesting that its role in intestinal inflammation is complex and still not fully understood. Therefore, by regulating IL-22 and IL-17 production, *Sutterella* represents a compelling subject for future research, especially in intestinal inflammatory conditions where immune dysregulation is a key driving factor. Moreover, we can speculate that *Sutterella* could lead to a favorable environment for the invasion of epithelial cells by other pathobionts. This hypothesis should be assessed, particularly as, in line with this, *Sutterella sp*. can also degrade IgA,^51^ which impacts the phagocytic capacity of human neutrophils,^52^ thus creating to profitable conditions to microbial infections.

Patients with UC exhibit an increased amount of *Sutterella* in their fecal microbiota^35^ ^,^ ^36^ and *S. wadsworthensis* is a marker of ineffective donor batches in the context of fecal microbiota transplantation in these patients.^53^ Our analysis of a Crohn's disease cohort revealed that patients undergoing a flare showed an increased relative abundance of *S. wadsworthensis* relative to healthy controls. While the abundance of the bacterium also correlates with disease duration, there is no association with medication exposure or surgical history. Even if we cannot exclude the effects of psychological, nutritional, and environmental aspects, our data suggest that the abundance of *Sutterella* is less likely to be artificially driven by external interventions and may rather reflect a link between *Sutterella* and the disease process itself. The bacterium could increase inflammation or sustain the disease over time by maintaining chronic inflammation. We can also speculate that there is a bidirectional interaction with the hypothesis that the host environment due to the disease, gradually promotes the presence of *Sutterella,* which in turn could enhance the inflammation.

Given the complex role of IL-22 and IL-17 in IBD, further work is needed to establish causal links in human disease and determine if *Sutterella* sp. may have a deleterious effect and represent a promising target for IBD therapy. This is also a potential biomarker for IBD diagnosis. The analysis of different clinical strains would be very informative from a mechanistic point of view, both at the bacterial and host levels. In exploring this promising hypothesis and identifying the implicated bio-active proteins, we will clarify the mechanisms of action and pave the way to develop innovative clinical strategies to control gut inflammation.

## Supplementary Material

Supplementary Material Supplementary_Figures.docx

## Data Availability

The 16S sequencing data derived from mouse samples have been deposited on Zenodo (zenodo.org/records/19554963). The sequencing data from the human cohort has been deposited on ENA (PRJEB114255), and the accession number will be added to the manuscript after acceptance. Any other data that support the findings of this study are available from the corresponding author upon reasonable request.
