# Supplementary material for: Inhibition of Aryl hydrocarbon receptor Interleukin-22 signaling and worsening of intestinal inflammation by Sutterella species
Source: Gut Microbes. 2026 Jun 21;18(1):2690688. doi: 10.1080/19490976.2026.2690688 (PMC13290079; doi:10.1080/19490976.2026.2690688)
Supplement: Supplementary Material — Supplementary_Figures.docx [file KGMI_A_2690688_SM5755.docx]

**Supplementary Figure 1. *Sutterella regulates IL-17 and IL-22 productions.*** Related to Figure 1

**(A)** Flow cytometry gating strategy used to identify gd T cells (gd TCR^+^ CD3^+^ CD4^neg^ CD8^neg^), CD4^+^ ab T cells (gd TCR^neg^ CD3^+^ CD4^+^ CD8^neg^), CD4^+^ CD3^neg^ cells (gd TCR^neg^ CD3^neg^ CD4^+^ CD8^neg^) and CD4^neg^ CD3^neg^ cells (gd TCR^neg^ CD3^neg^ CD4^neg^ CD8^neg^). **(B, C)** Percentage of IL-22 (**B**) and IL-17 (**C**) among gated CD4^+^ ab T cells, gd T cells, CD3^neg^ CD4^+^ and CD3^neg^ CD4^neg^ cells from colon obtained from untreated mice (control, CTL), neomycin (Neo)-, vancomycin (Vanco)– or neomycin+vancomycin (Mix)-treated mice. Cells were stimulated 4 hours with PMA + ionomycin + IL-1b + IL-23. **(D-F)** Correlation by Spearman r between percentage of IL-22 and IL-17-producing CD4^+^ ab T cells cells and *C. arthromitus* abondance in small intestine (**d**) and *Sutterellaceae* abondance in colon (**E, F**) from mice treated as in b.

Error bars are SEM from 5 to 10 mice per group (**B-F**). Significant differences were determined using Kruskal-Wallis test, *p < 0.05.

**Supplementary Figure 2. *Sutterella regulates IL-17 and IL-22 productions in vitro.*** Related to Figure 2

**(A)** Proportion of alive cells from pLN cultured 4h with BHI (black) or *S. wadworthensis* (Sw, blue) culture supernatant and stimulated with PMA + Ionomycin + IL-1b + IL-23 for the 3 last hours. **(B)** Intracellular analysis (top) and normalized geometric mean fluorescence intensity (bottom) of IL-22 (left) and IL-17 (right) expression by gated gd T cells and CD3^neg^ CD4^+^ cells from pLN cultured with BHI (black), *S. parvirubra* (Sp, red) culture supernatant. **(C)** Proportion of alive cells obtained as in B. **(D)** Intracellular analysis of IL-22 expression by gated gd T cells (left) and CD3^neg^ CD4^+^ cells (right) from pLN cultured with BHI (black), *S. wadworthensis* (Sw, blue) culture supernatant and stimulated as in A. pLN were collected from male C57BL/6J mice. **(E)** Intracellular analysis (left) and normalized geometric mean fluorescence intensity (right) of IFN-g expression by gated gd T cells from pLN cultured with BHI (black), Sp (red) culture supernatant. **(F)** Cells from LP of small intestine were cultured for 24h with BHI (black) or Sw (blue) or Sp (red) culture supernatant and stimulated with PMA + Ionomycin + IL-1b + IL-23. IL-6, TNF-a and IFN-g productions were measured in the supernatants. **(G)** HT29 cell line was cultured 24h with BHI (black) or Sw (blue) or Sp *(*red) culture supernatant in presence or absence of TNF-a. IL-8 production was measured in the supernatants.

Error bars are SEM with 13 mice (**A**), 7 mice (**B, C**), 7-8 mice (**D**), 4 mice (**E**), 3-4 mice (**F**) per group and 3 biological replicates (**G**). Significant differences were determined using Wilcoxon test, *P < 0.05.

**Supplementary Figure 3. *Sutterella regulates IL-17 and IL-22 productions in vivo.*** Related to Figure 3

**(A-D)** Intracellular analysis of IL-22 (**A**) and IL-17 (**C**) expression and normalized geometric mean fluorescence intensity for IL-22 (**B**) and IL17 (**D**) by gated ab CD4^+^ T cells, gd T cells, CD3^neg^ CD4^+^, CD3^neg^ CD4^neg^ cells from proximal small intestine obtained from PBS (control, black) or *S. wadsworthensis* (Sw, blue) -treated mice, and stimulated 3 hours with PMA + Ionomycin + IL-1b + IL-23. **(E)** Cells from pLN were cultured for 24h with PBS (black) or dead bacteria (MOI 10:1) (Sw, blue or *S. parvirubra,* Sp, red) and stimulated with PMA + Ionomycin + IL-1b + IL-23. IL-22 (left) and IL-17 (right) productions were measured in the supernatants. **(F-I)** Intracellular analysis of IL-22 (**F**) and IL-17 (**H**) expression and normalized geometric mean fluorescence intensity for IL-22 (**G**) and IL17 **(I)** by gated ab CD4^+^ T cells, gd T cells, CD3^neg^ CD4^+^, CD3^neg^ CD4^neg^ cells from proximal small intestine obtained from BHI (left), Sw (middle) or Sp (right) -treated mice, and stimulated as in A.

Error bars are SEM with 9-10 mice per group (**A-D, F-I**) and 7 mice (**E**). Significant differences were determined using Wilcoxon test, *P < 0.05, **P < 0.005 , ***P < 0.001.

**Supplementary Figure 4**. **Effect of *Sutterella in colitis*** Related to Figure 4

In all cases, mice were administrated with BHI, *S. wadsworthensis* (Sw) or *S. parvirubra* (Sp) culture supernatant and treated with DSS. **(A)** Weight and DAI of mice. **(B, C)** Normalized geometric mean fluorescence intensity for IL17 and IL-22 produced by gated ab CD4^+^ T (**B**) and CD3^neg^ CD4^+^ (**C, D**) cells, from colonic lamina propria stimulated for the 3 last hours with PMA + Ionomycin + IL-1b + IL-23.

Error bars are SEM with 19-20 mice (**A**), 10 or 20 mice (**B-D**) per group for each experiment. **P < 0.005 , by Mann-Whitney test.

**Supplementary Figure 5. *Sutterella regulates directly IL-22 producing cells.*** Related to Figure 5

**(A)** IL-22 mRNA quantification by qPCR in sorted ab CD4^+^ T cells cultured 4h with BHI (black) or *S. parvirubra* (Sp, red) culture supernatant and stimulated with PMA + Ionomycin + IL-1b + IL-23 for the 3 last hours. Mice were treated with DSS and ab CD4^+^ T cells were sorted from mesenteric LN. (**B**) Cells from pLN were cultured for 4 hours with BHI (black) or Sp (red) culture supernatant and stimulated with PMA + Ionomycin + IL-1b + IL-23 for the 3 last hours. IL-22 (left) and RORc (right) mRNA were quantified by qPCR. **(C, D)** Proportion of ROR-gt^+^ cells among total cells (**C**) or gd T cells (**D**) from LN cells cultured and stimulated as in B. **(E)** Proportion of IL-22^+^ cells among ROR-gt^+^ CD3^neg^ CD4^+^ cells from LN cells cultured and stimulated as in B. **(F)** Flow cytometric detection of intracellular pSTAT3 in gated gd T cells from pLN, cultured 2h with BHI or Sp (red) culture supernatant and stimulated with IL-23 for the last hour. Open and shaded areas indicate IL-23 treatment and controls, respectively. **(G)** Proportions of pSTAT3^+^ cells among gd T cells and CD3^neg^ CD4^+^ cells from pLN, cultured as in E. **(H)** Proportion of gd T cells from LP of small intestine of WT and AhR KO mice. **(I)** Intracellular analysis of IL-22 expression by gated gd T cells from small intestine cultured with BHI (black) or Sw (blue) culture supernatant and stimulated for the 3 last hours with PMA + Ionomycin + IL-1b + IL-23. **(J)** Proportion of gd T cells from pLN of WT and AhR KO mice. **(K)** Intracellular analysis of IL-22 expression by gated gd T cells from pLN of WT and AhR KO mice, cultured 4h with BHI (black) or *S. wadsworthensis* (Sw, blue) culture supernatant and stimulated with PMA + Ionomycin + IL-1b + IL-23 for the 3 last hours. **(L)** AhR reporter cell line activation without or with FICZ, in presence of BHI (black) or Sp (red) culture supernatant. **(M)** Adherent HT29 cells were cultured for 5 hours with BHI (black) or Sp (red) culture supernatant and stimulated with FICZ for the 4 last hours. Cyp1a1 (left) and AHRR (right) mRNA were quantified by qPCR.

Error bars are SEM with 9-11 mice **(A-D, G),** 8 mice (**F, J**), 4 mice (**H-I**), 7-8 mice (**K**), 3 biological replicates (**L**), 6 biological replicates **(M)** *P < 0.05, **P < 0.005; by Wilcoxon test.

***Supplementary Figure 6. Characteristics of the cohort.*** Related to Figure 6

***Supplementary Figure 7. A >3kDa protein from Sutterella regulates IL-22 productions.*** Related to Figure 7

**(A, B)** Proportion of alive cells from pLN cultured 4 hours and stimulated with PMA + Ionomycin + IL-1b + IL-23 for the 3 last hours. Cells were cultured with BHI or Sw culture supernatant (pretreated with proteinase K (PK) and/or pre-heated (Heat shock, HS) (**A**) or with <3 kDa and >3 kDa fractions (**B**) of BHI or Sw culture supernatant. (**C**) Proportion of alive cells from PBMC cultured 24h with <3 kDa and >3 kDa fractions of BHI or Sw culture supernatant and stimulated with anti-CD3 and anti-CD28.

Error bars are SEM from 8 mice (**A**), 13 mice (**B**) and 6 donors (**C**).
